# Exploring the role of metabolomics in kidney transplantation: a systematic review of the literature

**DOI:** 10.3389/fimmu.2025.1534875

**Published:** 2025-06-10

**Authors:** Cezar Valeriu Băluţă, Luminiţa Voroneanu, Ionuţ Nistor, Lucian Siriteanu, Andreea Simona Covic, Raluca Erika Irimie-Băluţă, Mehmet Kanbay, Adelina Vasilica Miron, Adrian Constantin Covic

**Affiliations:** ^1^ Grigore T. Popa University of Medicine and Pharmacy, Iaşi, Romania; ^2^ Spitalul Clinic Dr. C. I. Parhon, Iaşi, Romania; ^3^ Sfanta Parascheva Clinical Hospital for Infectious Diseases, Iaşi, Romania; ^4^ Department of Medicine, School of Medicine, Koc University, Istanbul, Türkiye

**Keywords:** kidney transplanation, systematic review, metabolomics, allograft function, kidney reject, metabolites

## Abstract

**Background:**

Serum creatinine and proteinuria remain the most frequently used test for monitoring allograft function. However, they are non-specific and insensitive markers. Metabolomics is an emerging field, dealing with the high-throughput identification and quantification of small molecules metabolites. We aimed to systematically review all available data regarding kidney transplantation and metabolomics.

**Methods:**

This is a systematic review evaluating metabolomic usage in kidney transplant patients. A comprehensive search was assembled in the time span extending from inception until March 2024 across MEDLINE (PubMed), *Embase* and Cochrane. In addition to the databases above, eligible citation were sought through the screening of ClinicalTrials.gov and Google Scholar. Two authors assessed potential citations for eligibility and quality and extracted all data.

**Results:**

A total of 57 articles were identified for inclusion (totaling 3821 patients), containing different methodologies and outcomes related to metabolic profiling. We aimed to offer support for finding new biomarkers that could aid in the evaluation of the kidney transplant patient, covering pathophysiological mechanisms and exploring avenues for personalized care.

**Conclussion:**

Our systematic review underlines the possible role of metabolomics in monitoring kidney transplant patients. By integrating data from numerous studies, we have detected possible new biomarkers that might transform the method we screen kidney transplant recipients.

## 1 Introduction

Chronic kidney disease (CKD) unfortunately remains a global healthcare burden (1), affecting more than 10% of the worldwide population. With a progressive evolution, its prevalence is higher in women, older individuals and some racial minorities (2). WHO reports that the number of CKD related deaths has risen, pushing CKD from the 13^th^ place to the 10^th^ place of top causes of death worldwide (3). Kidney transplantation is the gold-standard treatment for end stage kidney-disease (4). Despite the continuous evolution of immunosuppressive therapies which led to an improvement in the overall survival rates of both the allograft and the allograft recipients, long-term outcomes remain plagued by the persistence of allograft dysfunction (5), rejection risk (6) and opportunistic infections (7).

Serum creatinine and proteinuria remain the most frequently used test for monitoring allograft function. However, they are non-specific and insensitive markers which often cannot indicate early dysfunction (5). Some centers additionally use protocol kidney graft biopsies. While being the golden standard to evaluate graft structural and functional damage, kidney biopsies come with an associated procedure risk, the need for specialized pathologist and a higher cost, thus making them inconvenient and not readily available. Additionally, transplants can fail for many reasons other than acute graft rejection, including pre-operative organ stress, surgical complications and infectious complications, which cannot be predicted or diagnosed through graft biopsy. Immunosuppressive therapy can also damage the kidney directly and can also expose the patient to an increased risk of developing atherosclerosis, bone disease, chronic viral infections, diabetes, lymphoma or hypertension. The development and usage of novel techniques is the next logical step for an early diagnosis of a failing graft.

Metabolomics is an emerging field, that was developed in the early 2000, dealing with the high-throughput identification and quantification of small molecules metabolites in the metabolome. The metabolome is identified as the collection of small molecule metabolites, either endogenous or exogenous, which can be found in a cell, organ or organism; the terms metabonomics, metabolomics and metabolic profiling are interchangeable and can be used when describing the above method. Metabolic profiling may differ depending on the technique that is used, each strategy having to make a “trade off” between sensitivity, automated and high-throughput and metabolite identification (8).

Extending the clinical research in the field of genomic and proteomic methods might help identify different signature molecules that could be used to monitor kidney graft function and identify the risk of allograft disfunction earlier and more robustly (9). The kidney’s capability to concentrate or filter small molecule metabolites and toxins could make it possible to detect changes in the kidney function reflected in the levels of these elements, or even in changes that may appear to the kidney proteome or transcriptome (10).

The potential benefits of metabolomics in kidney transplantation are emerging (11). Therefore, a systematic evaluation of existing literature is a natural and essential next step to consolidate current knowledge and identify possible research gaps. In this review we aim to systematically assess the utilization of metabolomics in patients who have undergone kidney transplant, focusing on elucidating the role of metabolomics regarding graft function, rejection, post-transplant complications, opportunistic infections and immunosuppressive therapy. To achieve this objective, we outline our methodological approach below, detailing the search strategy, selection criteria and extraction methods employed in this systematic review.

## 2 Materials and method

In this systematic review, the research questions were formulated using the PICO (Population, Intervention, Comparison, Outcome) framework as recommended by Preferred Reporting Items for Systematic Reviews and Meta-Analyse (PRISMA) guidelines. We followed the updated guidelines outlined by the PRISMA; this involved addressing every aspect, including the methodology of our procedure and the collection and presentation of data. Two independent reviewers screened and assessed the studies for eligibility, resolving any discrepancies through discussion or a third senior reviewer. The protocol was approved and registered on the Open Science Framework (OSF) platform: https://osf.io/f2chb/?view_only=0b3445e250e641b5928013b55378ae80.

No modifications or amendments were made to the original registration protocol throughout the course of this study. The scope, inclusion and exclusion criteria, data extraction and analysis methods, outcome measures, and timeline as specified in the initial registration were followed without any alterations.

### 2.1 Data sources and search strategy

A comprehensive search was assembled in the time span extending from inception until March 2024 across MEDLINE (PubMed), *Embase* and Cochrane. In addition to the databases above, eligible citation were sought through the screening of ClinicalTrials.gov and Google Scholar. The search strategy was designed to capture all possible relevant studies published up to the date mentioned above, employing a combination of keywords related to metabolomics and kidney transplantation as follows: “kidney”, “transplantation” “metabolomics”, “NMR”, “nuclear magnetic resonance”, “kidney transplantation”, “liquid chromatography–mass spectrometry”, “LC/MS”. The search strategy is presented in Picture a.

### 2.2 Inclusion and exclusion criteria

Studies were included if they met the following criteria: (1) evaluated the metabolomic profile/metabolites in adult or pediatric patients with kidney transplant. (2) were available in English language, (3) evaluated the metabolites in biological products such as blood, urine, saliva or fecal matters. Exclusion criteria were also applied, and consisted of: (1) studies focusing solely on no transplant populations (2) populations with multiple organ transplant and (3) studies that involved an animal model.

As the metabolomic field is a relatively nascent and rapidly evolving domain characterized by numerous unknowns, we opted to include in our review not only full-text articles but abstract-only studies and conference abstracts, if they met the following criteria: (1) relevance to the topic – abstracts focusing on metabolomic techniques, biomarkers or outcome related to kidney transplant patients were deemed eligible, (2) availability of essential information – abstracts needed to provide sufficient details on the study design, patient characteristics, metabolomics method used and key findings; (3) currency of data – only abstracts reporting on recent research conducted were included. The decision to include these abstracts without full text was based on the consideration that the metabolomic technology is rapidly evolving and the inclusion of conference abstracts allowed for a comprehensive tour d’horizon on the subject.

### 2.3 Data extraction and synthesis

Each study was independently assessed by 2 reviewers; discrepancy between reviewers were resolved by consensus or by consulting a third senior reviewer when necessary. The accuracy of the extracted data was performed by cross-checking each entry by a second reviewer against the original articles. The following data were extracted from qualified papers that fulfilled the inclusion criteria: primary investigator, year of publication, population sample, number of samples, biological product that was evaluated, outcome, follow-up and kidney biopsy if these two were mentioned.

### 2.4 Quality assessment

Assessing the quality of the studies that were included in this review was realized with the Newcastle-Ottawa Scale (NOS); this tool is widely accepted as a method to evaluate and stratify the quality of methodology of included studies and the risk of bias in non-randomized studies. The scale evaluates 3 aspects of each study: the group selection, comparability and exposure. Each study receives points based upon fulfilling the criteria, with higher score indicating lower risk of bias. A detailed evaluation of each study based on NOS can be found in **Table 1**.

**Table 1:** Newcastle-Ottawa Scale.

Study	Case definition	Representativiness of the cases	Selection of Controls	Definition of controls	Comparability of cases& controls	Exposure asertainment	Ascertainment methods	Non-response Rate	Total
KIDNEY REJET									
1) Alkadi, M et al. (12)	NR	NR	NR	NR	NR	NR	NR	NR	NR
2) Banas, M et al. (13)	*	*	*	*	*	*	*	0	7
3) Banas, M et al. (14)	*	*	*	*	*	*	*	0	7
4) Dedinska, I et al. (15)	NR	NR	NR	NR	NR	NR	NR	NR	NR
5) Iwamoto, H et al. (16)	*	*	*	*	*	*	*	0	7
6) Iwamoto, H et al. (17)	*	*	*	*	*	*	*	0	7
7) Kalantari, S et al. (18)	*	*	*	*	*	*	*	0	7
8) Kim, S et al. (19)	*	*	*	*	*	*	*	0	7
9) Li X et al. (20)	*	*	*	*	*	*	*	0	7
10) Mao, Y et al. (21)	*	*	*	*	*	*	*	0	
11) Sigdel, T et al. (Poster – Abstract only) (22)	NR	NR	NR	NR	NR	NR	NR	NR	NR
12) Wang J et al. 23)	*	*	0	*	*	*	*	0	6
13) Zhao X et al. (24)	*	0	*	*	*	*	*	0	6
14) Zheng L et al. (25)	*	0	*	*	*	*	*	0	6
PEDIATRICS									
15) Sigdel T et al. (26)	*	*	*	*	*	*	*	0	7
16) Archdekin et al. (27)	*	0	*	*	*	*	*	0	6
17) Blydt-Hansen T et al. (28)	*	0	*	*	*	*	*	0	6
18) Blydt-Hansen T et al. (poster abstract Only) (29)	NR	NR	NR	NR	NR	NR	NR	NR	NR
19) Blydt-Hansen T et al. (30)	*	0	*	*	*	*	*	0	6
20) Taha K et al. (31)	*	0	*	*	*	*	*	0	6
IMMUNOSUPPRESSION									
21) Burghelea D et al. (32)	*	0	0	0	*	*	*	0	4
22) Dieme B et al. (33)	*	0	*	*	*	*	*	0	6
23) He X, et al. (34)	*	*	*	0	*	*	*	0	6
24) Kim C et al. (35)	*	*	*	*	*	*	*		
25) Kim S et al. (Poster – Abstract only) (36)	NR	NR	NR	NR	NR	NR	NR	NR	NR
26) Klepachi J et al. (37)									
27) Le Guellec C et al. (Poster – Abstract only) (38)	NR	NR	NR	NR	NR	NR	NR	NR	NR
28) Muhrez K et al. (Poster – Abstract only) (39)	NR	NR	NR	NR	NR	NR	NR	NR	NR
29) Xia T et al. (40)	*	0	*	*	*	*	*	0	6
30) Zhang F et al. (41)	*	0	*	*	*	*	*	0	6
ALLOGRAFT FUNCTION									
31) Baranovicova E et al. (42)	*	0	*	0	*	*	*	0	5
32) Bassi R et al. (43)	*	0	*	*	*	*	*	0	6
33) Blazquez-Navarro A et al. (44)	*	*	*	*	*	*	*	0	7
34) Colas L et al. (45)	*	*	*	*	*	*	*	*	8
35) Gagnebin Y et al. (Poster – Abstract only) (46)	NR	NR	NR	NR	NR	NR	NR	NR	
36) Ho J et al. (47)	*	*	*	0	*	*	*	0	6
37) Kim C et al. (Poster – Abstract Only) (48)	NR	NR	NR	NR	NR	NR	NR	NR	
38) Kouidhi S et al. (49)	*	0	*	*	*	*	*	0	6
39) Lan Y et al. (50)	*	*	0	*	*	*	*	0	6
40) Sigdel T et al. 41)Poster – Abstract only (51)	NR	NR	NR	NR	NR	NR	NR	NR	
42) Stenlund H et al. (52)	*	0	0	0	*	*	*	0	4
43) Suhre K et al. (53)	*	*	*	*	*	*	*	0	7
44) Suhre K et al. (54)	*	*	*	*	*	*	*	0	7
45) Verissimo T et al. (55)	*	0	*	*	*	*	*	0	6
46) Wang Z et al. (56)	*	0	*	*	*	*	*	0	6
47) Yozgat I et al. (57)	*	*	*	*	*	*	*	0	7
MISCELLANEOUS									
48) Calderisi, M et al. (58)	*	*	*	*	*	*	*	0	7
49) Iwamoto, H. et al. (16)	*	*	*	*	*	*	*	0	7
50) Kienana M. et al. (59)	*	0	*	*	*	*	*	0	6
51) Kouidhi S., et al. (60)	*	*	*	*	*	*	*	0	7
52) Li L., et al. (61)	*	*	*	*	*	*	*	0	7
53) Stanimirova I., et al. (62)	*	0	*	*	*	*	*	0	6
54) Gagnebin Y, et al., (63)	*	*	*	*	*	*	*	0	7
55) Liu R et al. (64)	*	*	*	*	*	*	*	0	7
56) Wang J et al. (65)	*	*	*	*	*	*	*	0	7
57) Dadhania D, et al. (Posteter – abstract only) (66)	NR	NR	NR	NR	NR	NR	NR	NR	

NR, not reported.The asterisk symbol * is used to indicate a point (representing a point) on the Newcastle-Ottawa Scale, according to the original methodology.

Subjective interpretations while applying the scale coupled with studies that were missing information (conference abstracts or studies with limited reporting) that led to insufficient data available to apply NOS criteria, could impact the overall quality assessment. Additional aspects were discussed in the limitation section.

## 3 Results

In this systematic review, the current literature regarding the use of metabolomics in kidney transplant patients was analyzed and summarized. Through a thorough search and selection process, a total of 57 articles were identified for inclusion (totaling a number of 3821 patients), containing different methodologies and outcomes related to metabolic profiling in the context of kidney transplant. Our review aimed to offer support for finding new potential biomarkers that could aid in the evaluation of the kidney transplant patient, covering at the same time pathophysiological mechanisms and exploring avenues for personalized patient care.

Selected studies varied in design and included cohort and case-control studies. The sample size ranged from small cohorts (10 patients) to larger ones (up to 310), revealing a diverse representation of kidney transplant recipients across demographic and clinical profiles. As stated, different techniques were employed including mass spectrometry, chromatography-based techniques, nuclear magnetic resonance spectroscopy. Additional information regarding the studies included in this study are provided in **Table 2** underlying relevant aspects including author details, publication year, study design, patient demographics, transplant details, immunosuppression information, hemodialysis vintage, and metabolomic utilized technique.

**Table 2:** General information.

Name, Year	Country	Population Size	Design type	Age - years	Sex(M/F)	Trx type	Time since trx	Immunotherapy	Tehnique	KB	HD vintage
KIDNEY REJET											
Alkadi M et all 2016 (Poster – Abstract Only) (12)	USA	105	Prospective	NR	NR	NR	NR	NR	LC-MS/MS and GC-MS	NR	NR
2. Banas M et all 2018 (13)	Germany	180	Retrospective	NR	NR	NR	NR	NR	NMR-spectroscopy	NR	NR
3. Banas M et all 2018 (14)	Germany	109	NR	NR	NR	NR	NR	NR	NMR – spectroscopy	NR	NR
4. Dedinska I et all 2022 (Poster – Abstract Only) (15)	Slovakia	55	NR	NR	NR	NR	NR	NR	NMR – spectroscopy	+	NR
5. Iwamoto H et all 2022 (Poster –Abstract Only) (67)	Japan	60 (51 KTR): -Donor - 9 -SKF - 19 -Impaired KF – 31	Retrospectiv	D – 64 (43 – 67) S – 49 (23-74) I – 47 (30-68)	D-3/6 S-11/8 I-22/9	Both	NR	P+MMF+: -TCA S– 11; I- 15 OR -CsA S– 8; I- 16	CE-MS	+	S 10- 16M I 20- 5,5M
6. Iwamoto H et all 2018 (Poster – Abstract Only) (17)	Japan	60 (51 KTR): -Donor - 9 -SKF - 19 -Impaired KF – 31	Retrospectiv	D – 64 (43 – 67) S – 49 (23-74) I – 47 (30-68	D-3/6 S-11/8 I-22/9	Both	NR	P+MMF+: -TCA S– 11; I- 15 OR -CsA S– 8; I- 16	CE-MS	+	S 10- 16M I 20- 5,5M
7. Kalantari S et all 2020 (18)	Iran	33 KTR: -TCMR-7 -SKF-11 -Cr rise - 15	Cross-sect retrospective	TCMR– 35,5±15 SKF- 37,4±13 Cr rise- 36,9±13,7	TCMR 6/1 SKF 9/6 Cr rise 10/1	Both	NR	P+MMF+: -TCA – -CsA-	H – NMR resonance	+	NR
8. Kim S et all 2019 (19)	S. Korea	31 KTR: -14-TCMR -17-SKF	Cross-sect multicenter	TCMR- 47±12 SKF- 44±14	TCMR-7/7 SKF-8/9	Both	TCMR-283 SKF-103 (days)	NR	LC-MS	+	NR
9. Li X et all 2022 (20)	China	60 KTR: 28 – AMR 32 - SKF	NR	AMR-33,29±7,1 SKF-37,31±8,5	AMR-25/3 SKF-26/6	NR	NR	P+MMF+TAC – 50 patients	LC-MS	+	NR
10. Mao Y et all 2008 (21)	China	37: -NM 15 -AR 22	Prospective	NM - 40±7.8 AR - 36.2±6.5	10/5&16/6	NR	NM: 30 AR: 4 – 730 (days)	P+MMF+: -CsA(13 NM–12 AR)OR -Rapa(0 NM–2 AR) OR -TCA(2 NM-8AR)	GC-MS	+	NR
11. Sigdel T et all 2018 (Poster – Abstract only) (22)	USA	NR	NR	NR	NR	NR	NR	NR	GC-MS	NR	NR
12. Wang J et all 2023 (23)	China	86: 30 KTR+AMR 35 KTR +SKF 21 ESRD	NR	AMR:33,7±7 SKF:38,6±8 ESRD:39,5±10	AMR:27/3 SKF:28/7 ERDS:15/6	NR	AMR: 5,3 SKF: 5,6 (years)	P+MMF+TAC	LC-MS	+	NR
13. Zhao X et all 2014 (24)	China	27: 11 – AR 16- Non AR	NR	AR:40,6±9,8 Non AR: ± 35,5±8,8	AR:8/3 NON AR:13/3	Csadaveric	NR	P+MMF+CsA	reversed-phase liquid chromatography (RPLC) and hydrophilic interaction chromatography (HILIC)	NR	NR
14. Zheng L et all 2018 (25)	China	30: 15-AR 15-Non AR	NR	AR:35,9±10 Non AR: 32,9±13	AR:12/3 Non AR:10/5	Both	AR: 0-3 years NonAR: 15 (days)	P+MMF+ AR:11CsA/4Tac NonAR:13CsA/2TAC	GC-MS	NR	NR
PEDIATRICS											
15. Sigdel T et all 2020 (26)	USA	310: AR - 106 STA - 111 IFTA - 71 BKVN – 22	NR	AR: 13±5 STA: 14±5 IFTA: 10±6 BKVN: 14±5	AR:49/57 STA:58/53 IFTA: 37/24 BKVN:16/6	Both	AR STA IFTA BKVN	NR	GC-MS	+	NR
16. Archdekin et all 2019 (27)	Canada	59	prospective	11.4±4.7	34/25	Both	NR	-PDN+MMF+: Tac/CsA MMF/AZA	DI-MS	+	1,4±1,4
17. Blydt-Hansen T et all 2014 (28)	Canada	57	NR	11,2±2,8	32/25	Both	NR	Tac/CsA MMF/AZA	LC-MS	+	NR
18. Blydy-Hansen T et all 2015 (Abstract only) (29)	Canada	57	NR	NR	NR	NR	NR	NR	NR	NR	NR
19. Blydt-Hansen T et all 2017 (30)	Canada	59	Prospective	11.4±4.7	34/25	Both	NR	-PDN+MMF+: Tac/CsA MMF/AZA	LC-MS	+	1,4±1,4
20. Taha K et all 2023 (31)	Canada+Mexico	52	NR	12±5,3	33/19	Both	1,6±2,5(years)	TAC/MMF/PDN	LC-MS	+	NR
OPORTUNISTIC INFECTIONS											
21. Dadhania D et all (Poster – Abstract only) (66)	NR	NR	NR	NR	NR	NR	NR	NR	NR	NR	NR
IMUNOSUPRESSION											
22. Burghelea D et all 2022 (32)	Romania	42- 23 low Tac 19 high Tac	NR	NR	NR	NR	NR	NR	NR	NR	NR
23. Dieme B et all 2014 (33)	France	35 12- CsA 23- Tac	NR	Tac:59 CsA:50	Tac:15/8 CsA:6/6	Cadaveric	De novo up until 180	P+MMF+ Csa:12 Tac:23	GC-MS	NR	NR
24. He X et all 2022 (34)	China	109 Norm:73 Low-Resp:16 High-Resp:20	Prospectiv	Norm:40 ±11 Low-resp:40± 11 High-resp:48± 8	Norm:49/24 Low-resp:12/4 High-resp:15/5	NR	7 Days	P+MMF+TAC	UPLC/Q-TOF-MS	NR	NR
25. Kim CD et all 2010 (35)	S Korea	57: 27CsA grp 30Tac grp	Prospectiv	NR	CsA:22/5 Tac:21/9	both	1>90>180	P+MMF+ -TAC 30 -CsA 27	1H-NMR	NR	NR
26. Kim S et all 2013(Poster – Abstract only) (36)	USA	20: TAC – 8 Sir – 3 HC - 9	Observational	NR	NR	NR	NR	TAC Sir	NR	NR	NR
27. Klepachi J et all 2016 (Poster – Abstract only) (37)	SUA	120: 306 EVR+lowTAC 304 MMF+stdTAC	Non inferiority	EVR grp: 50 MMF grp: 48,4	EVR grp:205/101 MMF grp:202/102	Both	De novo	EVR+ low TAC MMF+ sdt TAC	LC-MS/MS	+	NR
28. Le Guellec C et all 2013(Poster – Abstract only) (38)	France	47	NR	NR	NR	NR	NR	TAC CsA	1H-NMR+ GC/MS	NR	NR
29. Muhrez K et all 2014 (Poster-Abstract only) (39)	France	38: Tac – 25 CsA – 13	NR	NR	NR	NR	7days>90>365	PDN+MMF+ Tac – 25 CsA- 13	1H-NMR	NR	NR
30. Xia T et all 2018 (40)	China	22: HC – 11 KTR -11	descriptive	HC – 48 KTR – 38	HC – 166/122 KTR – 6/5	NR	NR	PDN+MMF+TAC	HPLC-MS/MS	NR	NR
31. Zhang F et all 2018 (41)	China	66: 24 HC 12 NON-AKI KTR 30 AKI KTR	Retrospective review	HC – 24,5±3 NON-AKI - 402±14,4 AKI – 36,2±8,8	HC:13/11 NON-AKI:8/4 AKI: 15/15	Living	NR	PDN+MMF+TAC	UHPLC-MS/MS	NR	NR
ALLOGRAFT FUNCTION											
32. Baranovicova E et all 2022 (42)	Slovakia	55: DIVIDED BY eGFR value: 1- 1 2- 21 3- 21 4- 10 5- 2	Observational	Stg: 1- 41 2- 48,4 3- 56.8 4- 54.5 5- 42,48	Stg: 1- 1/0 2- 11/10 3- 13/8 4- 5/5 5- 2/0	Cadaveric	NR	PDN+MMF+TAC	NMR-spectroscopy	+	NR
33. Bassi R et all 2017 (43)	USA	50: 10 – HC T1 T2 T3	Observational	T1 - 56 T2 - 62 T3 - 55	NR	Both	>180 days	PDN+MMF+Tac-15 Tac+Rapa -2 Tac+Aza – 2 CsA+Aza – 2 CsA+MMF – 1 Rapa+PDN – 2 Tac+PDN - 3 CsA+PDN – 3 MMF – 4 Tac- 5 CsA - 1	LC MS/MS	NR	T1 – 56m T2 – 53M T3 – 78M
34. Blazquez-Navarro A et all 2022 (44)	Germany	376: T1 - 139 T2 - 125 T3 - 112	Prospective-observational	55	246/130	Both	NR	PDN+MMF+Tac T1 T2&T3 – stop PDN 8th day	1H-NMR	NR	NR
35. Colas L et all 2022 (45)	France	56: 14 - HC 16 –Patients with no immunosuppression (TOL) 5 – AMR 13 – Patient with minimally immunosuppression (MIS) 8 – Normal Histology(STA)	retrospective	HC TOL AMR MIS STA -	HC- 7/7 TOL- 13/3 AMR- 3/2 MIS-11/2 STA-6/2	Both	TOL-176-433 AMR-160-375 MIS-60-336 STA-168-236 months	PDN-STA (1), MIS(11), AMR(1) MMF-STA(7), MIS(9), AMR(3) CNI- STA(5), AMR(4) mTOR I- STA(3), AMR(1)	LC/MS	+	NR
36. Gagnebin Y et all 2019 (Poster – Abstract only) (46)	NR	NR	NR	NR	NR	NR	NR	NR	NR	NR	NR
37. Ho J et all 2016 (47)	Canada	113 – KTR: -No inflammation-66(normal 33, IFTA 33) -Mild inflammation- 58(IFTA 10, Borderline 18, Subclinic 30) -Severe inflammation- Clinic - 13	Retrospective - observational	No inflammation: -Normal-44±12 -IFTA - 46±12 Mild inflammation: -IFTA 46±12 -Borderline 42±12 -Subclinical 43±11 Severe inflammation: Clinical 43±11	No inflammation 43/23 Mild inflammation Severe inflammation	Both	NR	PDN+MMF+ Tac -74 CsA - 39	Direct-Flow Injection/MS	+	NR
38. Kim C et all 2017 (Poster – Abstract Only) (48)	Korea	34 total KTR: 24 - LGS 10 – Chronic AMR	NR	NR	NR	NR	NR	NR	NR	NR	NR
39. Kouidhi S et all 2021 (49)	Tunis	60: 20-HC 40-KTR with SKF	NR	HC- 44±6 KTR - 42±5	HC: 10/10 KTR: 27/13	Both	3-264 MONTHS	1 AZA+Tac PDN+MMF+Tac-21 PDN+MMF+CsA 3 PDN+mTOR I -2 PDN+Tac – 2	GC-MS	NR	NR
40. Lan Y et all 2023 (50)	CHINA	100: eGFR>60 – 53(T1) eGFR<60 – 47(T2)	NR	T1- 37,9±10 T2 – 42,7±8	T1- 40/13 T2 – 36/11	NR	>3 months	T1 – MMF(47), CsA (10), TaC(43)	LC-MS	NR	NR
41. Sigdel T et all 2014 (Poster – Abstract only) (51)	NR	NR	NR	NR	NR	NR	Between 1 and 15 days	NR	NR	NR	NR
42. Stenlund H et all 2009 (52)	Sweden	19	Obervational	NR	NR	NR	Between 1 and 8 days	NR	1H-NMR	NR	NR
43. Suhre K et all 2021 (53)	USA	153 KTR: Normal biopsy - 29 ATI - 49 PVAN - 32 Mixed rejection - 14 AMR - 16 ACR - 22	NR	Normal biopsy - ATI – 51,2 PVAN – 56,9 Mixed rejection – 40,9 AMR – 41,8 ACR – 50,5	99/54 Normal biopsy – 18/11 ATI – 34/15 PVAN – 21/11 Mixed rejection – 10/4 AMR – 8/8 ACR – 15/7	Both	Normal biopsy – 2,8-42,8 ATI – 0,2-109,2 PVAN – 2,7-70M Mixed rejection – 0,13-146M AMR – 0,4-162 M ACR - 0,2-182M	NR	NR	+	NR
44. Suhre K et all 2016 (54)	USA	185 – KTR ACR – 36 No rejection - 149	NR	ACR – 45,1 No ACR – 47,3	127/58 ACR: 27/9 No ACR: 100/49	Both	NR	NR	LC/MS, GC/MS	+	NR
45. Verissimo T et all 2022 (55)	Switerland	42 -KTR 1 year GFR >51 - 21 1 year GFR <51 – 21	NR	1 year GFR< 51 - 50 1 year GFR >51 – 54,6	1 year GFR< 51 – 14/7 1 year GFR >51 – 13/8	Both	>1 year	PDN+MMF+ Tac 15	NR	+	NR
46. Wang Z et all 2017 (56)	China	36: DGF - 11 IGF - 25	NR	DGF – 39,15±1,42 IGF – 37,94 ±1,93	DGF – 10/1 IGF – 23/2	Cadavaeric	Graft perfusion pre TRX	NR	1h-NMR	NR	NR
47. Yozgat I et all 2023 (57)	Turkey	131: -S1 – 53 (eGFR>60ml/min) -S2 - 56 (30<eGFR<60 ml/min) -S3 – 22 (eGFR<30ml/min)	NR	S1 – 47,5±13,26 S2 – 46,6±10,51 S3 – 52±14,31	S1 – 35/18 S2 – 40/16 S3 – 9/13	Both	NR	NR	NR	NR	NR
MISCELLANEOUS											
48. Calderisi M et al. – 2013 (58)	Italy	15	NR	NR	9/6	NR	>1 day	NR	1H-NMR	NR	NR
49. Iwamoto H et al. – 2022 (16)	Japan	59: Impaired KF – 31 Stable KF – 19 Donors - 9	NR	Donors – 64 Stable KF – 49 Impaired KF - 47	Donors-3/6 Impaired KF-22/9 Stable KF -11/8	Both	NR	Stable KF – Tac 11, MMF 19; Impaired KF – Tac 15, MMF 30	GC/MS LC/MS	NR	Stable KF – 16 M Impaired KF – 5,5
50. Kienana M et al. – 2015 (59)	France	KTR: 38	NR	Tac grp: 59 CsA grp: 50	22/16	NR	>7 days	PDN+MMF+ Tac: 15 CsA: 13	1H-NMR GC-MS	NR	NR
51. Kouidhi S et al. 2021 (60)	Tunisia	60: HC: 20 KTR – 40	NR	HC: 44 KTR: 42	HC: 10/10 KTR: 28/12	NR	6years (mean)	PDN+MMF+Tac	GC-MS	NR	NR
52. Li L et al. 2013 (61)	China	48: 20- KTR 28- HC	NR	KTR: 39,15±8,29 HC: 37,74±8,45	KTR: 7/13 HC: 10/18	Both	After KTR	PDN+AZA+Tac	1H-NMR	NR	13,4±1,6 M
53. Stanimirova I et al. 2020 (62)	Poland	KTR: 19	NR	KTR: 55,5±13,6	13/6	NR	Pre and post KTR	NR	1H-NMR	NR	NR
54. Gagnebin Y et all, 2020 (63)	Switerland	66: 24 – Donors 42 – TRX	Prospective	Donors: 52,8±9,5 Trx - 518±13,1	Donors: 7/17 TRX: 35/7	NR	Day 1	NR	LC-MS	NR	NR
55. Liu R, et all 2023 (64)	USA	Deceased donor: 147 Recipient: 190	NR	Donors: 47±14 Recipient: 57±14	Donors: 93/54 Recipeint: 124/66	Cadaveric	Hypotermic machine perfusion – during TRX surgery	NR	MS	+	NR
56. Ma C, et all 2021 (Abstract only) (68)	NR	NR	NR	NR	NR	NR	NR	NR	NR	NR	NR
57. Wang J et all 2011 (65)	China	10: 5 KTR 5 Donors	Observational prospective	KTR: 33,4 Donors: 40,4	Donors – 1/4 KTR – 4/1	Living	Between 1-35 days	PDN+MMF+ CsA – 3 Tac - 2	MALDI-MS	NR	NR

NR, not represented; AR, Acute rejection; CE-MS, capillary electrophoresis, mass spectometry; LC-MS, liquid chromatography, mass spectometry; NMR, nuclear magnetic resonance; KTR, kidney transplant reciepents SKF, stable kidney function; Cr, creatinine; NR, not reported; TCMR, T-Cell mediated rejection; AMR, antibody mediated rejection; ESRD, end stage renal disease; HC, healthy controls; Rapa, Rapamicin; Aza, Azathioprine; PDN, prednisone; MMF, mycophenolate mofetil; PVAN, polyomavirus-associated nephropathy; AZA, Azaathioprine; MALDI-MS, matrix-assisted laser desorption/ionization, mass spectrometry; DGF, delayed graft function; IGF, immediate graft function; IFTA, interstitial fibrosis and tubular atrophy; BKVN, BK virus nephropathy; LGS, long-term good survival; EVR, everolimus; TAC, tacrolimus; Cr, creatinine.

The studies included were divided and grouped based on the outcome that was evaluated: allograft function, immunosuppression, the vast domain of graft rejection, miscellaneous, pediatric patients, opportunistic infections.

Across studies, consistent findings showed alterations in various metabolite profiles. Dysregulation in pathways related to lipid, amino acid and energy metabolism reflect the complex interaction between the transplanted organ, the host, medication and other factors.

*The allograft function was the main outcome of 16 studies* (totaling 1410 patients) (42–57). Details about the outcome are reported in the **Table 3**. These studies reports that certain metabolites from plasma/urine/feces alone or in combination could be used as a potential prediction tool to monitor kidney function. Studies involved a range of patient’s size from relatively small (19 pts) to larger cohorts (almost 400 pts) and collected blood, urine or fecal samples in order to establish the metabolomic profile. Different techniques were used; mass spectrometry and nuclear magnetic resonance remained the most commonly used. Baranicova et al. noted a higher *glutamine* plasma level in posttransplant patients undergoing *acute cellular rejection and acute antibody-mediated rejections* compared to patients without rejection. Another metabolite involved was *histidine*, whose plasma levels increased with serum creatinine and decreased with eGFR (42). In an elegant study, Wang et al. performed a graft perfusion with hypertronic citrate adenine II prior to the transplantation surgery. By testing 15 ml of the perfusate from the initial outflow of the allograft renal vein after transplantation, they revealed over 30 metabolites correlated with delayed graft function. Among them, *citrate, a-glucose, betaine and taurine* were underlined as having a significant correlation with delayed graft function (56).

**Table 3:** Studies evaluating allograft function.

NR Author, Date	Participants	Specimen, No	Outcome	Results	Follow-up	KB
1. Baranovicova E. et al. (42)	55 of which: 35 – controls 10 - AMR 10 - TCMR	Blood - 55	Relative levels of basal plasma metabolites detectable by NMR spectroscopy.	Results imply a quantitative relationship between restricted renal function, insufficient hydroxylation of phenylalanine to tyrosine, lowered renal glutamine utilization, shifted nitrogen balance, and other alterations that are not related exclusively to the metabolism of the kidney.	50 months	Yes
2. Bassi R. et al. (43)	40 – various degrees of graft dysfunction (T1 = 56–108 ml/min; T2 = 46–55 ml/min; and T3 = 21–39 ml/min) 10 - HC	Blood+Urine	Profile of metabolomic abnormalities induced by the progressive reduction of kidney function	LC-MS/MS analysis revealed a dose-response association between GFR and serum concentration of tryptophan, glutamine, dimethylarginine isomers and short-chain acylcarnitines The same association was found between GFR and urinary levels of histidine, DOPA, dopamine, carnosine, dimethylarginine isomers,	6 months	NR
3. Blazquez-Navarro A. et al. (44)	376	958	Aimed to build an early predictor of an established long-term outcomes marker.	Found evidence of an association of eGFR with the cytokine SCF and the urine metabolomic profile.	NR	NR
4. Colas L. et al. (45)	56 of which: 14 - HC 16 –patients with no immunosuppression 5 – AMR 13 – patient with minimally immunosuppression 8 – normal Histology	Urine - 56	Evaluation of the metabolomic signature of patients with spontaneous operational tolerance	We could identify a specific urinary metabolomic profile strongly driven by the up-regulation of the tryptophanderived metabolites; kynurenine, kynurenic acid and tryptamine independent of any immunosuppressive drugs and serum creatinine level in spontaneous tolerant patients.	NR	Yes
5. Gagnebin Y. et al. (46)	66 of which 24 – living donors 42 – KTR	Blood – 1- before KT 1 - W1 1 - M1	The benefits of metabolomics in the transplant patients and voluntary donors monitoring	More than 250 plasma metabolites were identified using multi-platform analytical setup and were monitored using two specific AMOPLS models for graft patients and donor volunteers.	NR	NR
6. Ho J. et al. (47)	113 patients	137 KB of which: 66 – no rejet 58 – mild rejet 13 – severe rejet 113 Urine samples	Characterize urinary metabolomics for detection of cellular inflammation. Determine if adding urinary chemokine ligand 10 (CXCL10) improves the overall diagnostic discrimination.	Urinary metabolomics can noninvasively discriminate noninflamed renal allografts from those with subclinical and clinical inflammation. The addition of urine CXCL10 had a modest but significant effect on overall diagnostic performance. These data suggest that urinary metabolomics and CXCL10 may be useful for noninvasive monitoring of alloimmune inflammation in renal transplant patients.	NR	Yes
7. Kim C. D. et al. (48)	34 of which 24- long term good survival 10 - CAMR	Blood - 34	To develop biomarkers that can predict long-term survival on KTRs through metabolomics	Found serum metabolites which differ between long term good survival and CAMR groups.	NR	Yes
8. Kouidhi S. et al. (49)	60 of which 40 – KTR 20 – HC	Fecal - 60	The fecal metabolic profile in immunosuppressive patients vs HC	The metabolomic signature showed dramatic changes in response to immunosuppressive therapy; the model showed a clear trend of group clustering between the kidney transplant group and the control healthy group, with both groups having unique metabolome profiles	NR	NR
9. Lan Y. et al. (50)	100 of which 53 – eGFR >60ml/min(CKD G1-2T) 47 – eGFR <60 but > 30 ml/min(CKD G3T)	Fecal	Analyze fecal metabolic profiles to investigate the alterations of gut microbiome in CKd-t	Gut microbiome and metabolites in the progression of CKD-T display some unique distribution and expression characteristics. The composition of the gut microbiome and their metabolites appears to be different between patients with CKD G3T and those with CKD G1-2T.	NR	NR
10. Sigdel T. et al. (51)	340	Urine 340 – 106 – AR 111 – stable KF 81 – chronic graft injury 22- BKVN 20 - HC	A panel of urine metabolites to detect transplant injures	152 metabolites were differentially present. A panel of 59 metabolites was able to distinguish AR urine from STA in a training set with 87% sensitivity and 93% specificity.	NR	Yes
11. Stenlund H. et al. (52)	19	Urine – 1 probe per day for 15 days after KT	Identifying a profile reflecting biochemical changes that occur in the first two weeks following a kidney transplant.	The metabolite profiles differed widely from each other and changed substantially over time. For each patient, samples were grouped and assigned into before and after graft functioning. Shifts were observed at the regions that corresponds to methyl-groups in creatinine. This finding confirms that creatinine is the most significant metabolic marker for kidney function after a transplant.	NR	NR
12. Suhre K. et al. (54)	241	Urine - 1516	Metabolites that are measured in the urine may inform about kidney function and health status.	The ratio of the concentrations of the metabolites 3-sialyllactose to xanthosine (3SL/X) in the urine supernatant was strongly associated with ACR and this ratio had the highest increase in the strength of association without regards of age, gender or ethnicity.	NR	Yes
13. Suhre K et al. (53)	153 of which AMR – 16 TCMR – 22 Mixed rej – 14 ATI – 51 PVAN - 36	Urine - 192	Metabolites that are measured in the urine may inform about kidney function and health status.	Non-targeted metabolomics data for 674 metabolites and 577 unidentified molecules were analyzed. Univariate and multivariate analyses identified metabolite signatures for kidney allograft rejection.	NR	Yes
14. Verissimo T. et al., (55)	42	Kidney reperfusion biopsies	The estimated metabolites abundance was further used to predict the one-year allograft renal function	Estimated the renal metabolome, and its abundance was used to predict the renal function within the first year of transplantation through a random forest machine learning algorithm. An optimal model was proposed and it accurately predicted the one-year eGFR.	NR	yes
15. Wang Z. et al., (56)	DGF – 11 IGF - 25	Perfusate samples from kidney allografts	Collected perfusate samples from kidney allografts prior to transplantation, and used metabolomic analysis	Identified 37 metabolites; among these, 4 important endogenous metabolites, citrate, a-glucose, betaine and taurine, were significantly associated with the occurrence of DGF.	NR	NR
16. Yozgat I. et al (57)	131 of which: 53 - eGFR>60ml/min(S1) 56 – 30<eGFR<60ml/min(S2) 22 – eGFR<30ml/min	Urine	Identify specific eGFR based biomarkers to monitor indifiduals with different levels of post-transplantation graft dysfunction.	Metabolites that were significantly altered within three groups of kidney transplant recipients:4,5-Dihydroorotic acid, N2- Succinyl-L-glutamic acid 5-semialdehyde, Valyl-Arginine, Pantothenic acid, L-phenylalanyl-L-hydroxyproline, MG(0:0/24:0/0:0), QYNAD and 12-Hydroxy-13-O-D-glucuronoside-octadec-9Z-enoate. The ratio of 4,5-Dihydroorotic acid to Pantothenic acid can be used to monitor kidney function.	NR	NR

AMR, Antibody mediated rejection; TCMR, T-cell mediated rejection; CG, control group; BCKA, branched-chain keto acids; BCAA, branched-chain amino acids; Cre, Creatinine; eGFR, estimated Glomerular-filtration rate; HC, Healthy Controls; KF, Kidney function; Cre, creatinine; KTR, kidney transplat recipients; W, week; M, month; KT, kidney transplant; CAMR, chronic antibody mediated rejection; CKD, chronic kidney disease; AR, acute rejection; BKVN, BK Virus nephropathy; ATI, acute tublar injury; PVAN, polyomavirus-associated nephropathy; DGF, delayed graft function; IGF, immediate graft function; LGS, long-term good survival; NR, not reported.

Focusing on the progression of metabolomic profile in the immediate posttransplant period (up to 15 days), Stenlund et al. illustrate the dynamic nature of metabolomic changes following transplantation (52). Their conclusion was that the metabolite profiles vary among individuals and change over time. Changes that become apparent in metabolites up to 6 months post-transplant (that reflect key metabolic changes in the accommodation process) were also evaluated by Stanimirova I et al., 19 patients with normal recovery post-transplant were included. Higher levels of *valine, alanine, glutamine, methionine, GPC+APC, mannitol, glucose and lower levels of creatinine, citrate, myo-inositol, lactate, histidine, hippurate and adenine* were identified in the post-transplant serum of patients when compared to pre-transplant. Moreover, the minimum number of metabolites that can be used to monitor renal function includes hippurate, mannitol and alanine. Specifically, it was found that the level of *hippurate* was more sensitive to changes in renal function, while monitoring the creatinine is appropriate for indicating large changes in renal function such as those before/after graft surgery. The most difficult distinction was for the metabolic state in the intermediate period after transplantation (T1 and T2) (62).

In patients with *progressive reduction of kidney function*, Bassi et al. showed a gradual elevation of *glutamine* levels compared to patients with preserved kidney function, suggesting a potential link between glutamine metabolism and progressive kidney dysfunction posttransplant. Additionally, an overall reduction in urinary levels of amino acids and biogenic amines in patients with poor graft function was also described. Among biogenic amines, the urinary concentration of carnosine was reduced in patients with a failing graft (43).

14 studies involving 813 patients had *kidney graft rejection as a main outcome* (12–15, 17–25, 67), with differentiation between antibody mediated rejection (AMR) and T cell mediated rejection (TCMR). **Table 1** presents detailed information about the outcome. **Table 4** outlines the metabolites that were identified and the trend they followed depending the type of rejection.

**Table 4:** Metabolite trends associated with different types of renal allograft rejection.

Common Name	HMDB - ID	Type of rejet		Hits	Type/Class
		**AMR **	**TCMR**		
Xanthosine	HMDB0000299	↑ urine (12)	n/a	1	Purine-nucleosides
Quinolinate	HMDB0000232	↑ urine (12)	n/a	1	Pyridinecarboxylic acids
3-sialyllactose	HMDB0000825	↑ urine (12)	n/a	1	n-acylneuramic acid
Lactate	HMDB0000190	↓ plasma (42)	↑plasma (15)	2	Alpha hydroxy acids
Glutamine	HMDB0000641	↑plasma (15, 42)	↑plasma (15, 42)	1	l-alpha-amino acids
Tyrosine	HMDB0000158	↓ plasma (15, 42)	→	2	Alpha-amino acid
3-indoxyl sulfate	HMDB0000682	n/a	↑plasma (67) ↑urine (67)	1	Arylsulfates
Gluconate	HMDB0000625	n/a	↑plasma (67)	1	Sugar acid
*N*,*N*-dimethylglycine	HMDB0000092	n/a	↓ plasma (67)	1	Alpha-amino acid
Choline	HMDB0000097	n/a	↓ plasma (67)	1	Cholines
Threonine	HMDB0000167	n/a	↓ plasma (67)	2	Alpha-amino acid
Methionine	HMDB0000696	n/a	↓ plasma (67)	1	Alpha-amino acid
S-adenosyl methionine	HMDB0001185	n/a	↑urine (67)	1	5'-deoxy-5'-thionucleosides
Citrate	HMDB0000094	n/a	↑plasma (67)	1	Tricarboxylic acid
Hyroxyproline	HMDB0000725	n/a	↑plasma (67)	1	Proline and derivatives
Aspartic acid	HMDB0006483	n/a	↑plasma (67)	1	Aspartic acid and derivatives
Nicotinamide adenine dinucleotide (NAD)	HMDB0000902	n/a	↑urine (18)	1	Dinucleotides
Cholesterol sulfate	HMDB0000653	n/a	↑urine (18)	1	Cholesterols
1-methylnicotinamide	HMDB0000699	n/a	↑urine (18)	1	Nicotinamides
Nicotinic acid	HMDB0001488	n/a	↑urine (18)	1	Pyridinecarboxylic acids
Gamma aminobutyric acid	HMDB0000112	n/a	↑urine (18)	1	Gamma amino acid
Nicotinamide adenine dinucleotide phosphate (NADP)	HMDB0000217	n/a	↑urine (18)	1	Catechols
Homocysteine	HMDB0000742	n/a	↓ urine (18)	1	Alpha-amino acid
Proline	HMDB0000162	↑plasma (42)	↑urine (18), ↑plasma (42)	2	Alpha-amino acid
Spermidine	HMDB0001257	n/a	↑urine (18)	1	Dialkylamines
Guanidoacetic acid	HMDB00128	n/a	↑urine (19)	1	Alpha amino acid
Methylimidazoleacetic acid	HMDB02820	n/a	↑urine (19)	1	Imidazolyl carboxylic acid
Dopamine	HMDB00073	n/a	↑urine (19)	1	Catecholamine
4-Guanidinobutyric acid	HMDB03464	n/a	↓urine (19)	1	Gamma amino acid
*L*-Tryptophan	HMDB0000929	n/a	↓urine (19)	1	Indolyl carboxylic acid
Xanthine	HMDB00292	n/a	↑urine (19)	1	Xanthine
*N*-acetyl-L-histidine	HMDB0032055	↓feces (20)	n/a	2	Histidine and derivative
3b-Hydroxy-5-cholenoic acid	HMDB0000308	↓feces (20)	n/a	2	Monohydroxy bile acid
Ferulic acid	HMDB0000954	↓feces (20)	n/a	1	Hydroxycinnamic acid
2-isopropylmalic acid	HMDB0000402	↓feces (20)	n/a	1	hydroxy fatty acid
N6, N6, N6-trimethyl-L-lysine	HMDB0001325	↓feces (20)	n/a	1	l-alpha-amino acid
alpha-ketoglutarate	HMDB0000208	↑feces (20)	n/a	1	gamma-keto acid
phenol	HMDB0000228	↑feces (20)	n/a	1	1-hydroxy-4-unsubstituted benzenoid
N1-methyl-2-pyridone-5-carboxamide	HMDB0004193	↑feces (20)	n/a	1	nicotinamide
Taurocholate	HMDB0257923	↑feces (20)	n/a	1	taurinated bile acid
Phenylalanine	HMDB0000159	↑plasma (21, 42)		1	Phenylalanine and derivates
Serine	HMDB0000187	↑plasma (21) ↑urine (22)		2	Serine and derivates
Glycine	HMDB0000123	↑plasma (21) ↑urine (22)		2	Alpha amino acid
Threonine	HMDB0000167	↑plasma (21) ↑urine (22)		2	Alpha-amino acid
Valine	HMDB0000883	↓plasma (21) , ↓plasma TCMR (42)		2	Valine and derivatives
Lysine	HMDB0000182	↓plasma (21)		1	alpha-amino acid
Leucine	HMDB0000687	↓plasma (21) , ↓plasma TCMR (42)		2	Leucine and derivative
Alanine	HMDB0001310	↑plasma (21)		1	Alanine and derivatives
Galactose oxime	–	↑plasma (21)		1	–
Glucose	HMDB0000122	↑plasma (21) ↑urine (25)		2	hexoses
Fructose	HMDB0000660	↑plasma (21) , ↓urine (25)		2	Monosaccharide
1,2,3-propanetricarboxylic acid	HMDB0031193	↑plasma (21)		1	Tricarboxylic acids and derivative
2,3,4-Trihydroxybutyric acid	HMDB0245425	↑plasma (21)		1	Sugar acids and derivatives
Hexadecanoic acid	HMDB0010734	↑plasma (21)		1	Long-chain fatty acids
Octadecanoic acid	HMDB0010737	↑plasma (21) , ↑urine (25)		2	Long-chain fatty acids
Oleic acid	HMDB0000207	↑plasma (21)		1	long-chain fatty acids
Aminomalonic acid	HMDB0001147	↓plasma (21)		1	alpha amino acids
Tetradecanoic acid	HMDB0000806	↓plasma (21)		1	long-chain fatty acids
Lactate	HMDB0000190	↑plasma (21)		2	Alpha hydroxy acids
Myo-inositol	HMDB0000211	↑plasma (21), ↑urine (25)		2	Cyclohexanols
1,7-dimethyluric acid	HMDB0011103	↑plasma (24)		1	Xanthines
Taurochenodeoxycholic acid	HMDB0000951	↑plasma (24)		1	Bile acid
Glycochenodeoxycholic acid	HMDB0000637	↑plasma (24)		1	Bile acid
Kynurenine	HMDB0000684	↓ plasma (24)		1	alkyl-phenylketones
PUFAs	Multiple representatives of the classes.	↓ plasma (24)		1	
Sphingomyelins					
Phosphatidylcholines					
Lysophosphatidylethanolamine					
Lysophosphatidylcholines					
Threitol	HMDB0004136	↑urine (25)		1	sugar alcohol
Phosphate	HMDB0001429	↑urine (25)		1	non-metal phosphates
Xylono-1,5-lactone	HMDB0011676	↑urine (25)		1	delta valerolactones
Ribonic acid	HMDB0000867	↑urine (25)		1	sugar acids and derivatives
Xylitol	HMDB0002917	↑urine (25)		1	sugar alcohols
2,3-dihydroxybutanoic acid	HMDB0245394	↑urine (25)		1	sugar acids and derivatives
Glucitol	HMDB0000247	↑urine (25)		1	sugar alcohols
3-hydroxyisovaleric acid	HMDB0000754	↓urine (25)		1	hydroxy fatty acid
Glycolic acid	HMDB0000115	↓urine (25)		1	alpha hydroxy acid
BKVN	HMDB - ID		Hits	Type/Class
Galactose metabolism	n/a	↑urine (22)	1	n/a
Gluthatione metabolism	n/a	↑urine (22)	1	n/a
β-alanine metabolism	n/a	↑urine (22)	1	n/a
AMR Compared to ESKD and KT-SKF	HMDB - ID	Biological product	Hits	Type/Class
N-Palmitoylsphingosine	HMDB0004949	↑feces (23)	1	n/a
Erucamide	HMDB0244507	↑ feces (23)	1	Fatty amides
3b-Hydroxy-5-cholenoic acid	HMDB0000308	↓ feces (23)	1	Monohydroxy bile acid
N-Acetyl-L-Histidine	HMDB0032055	↓ feces (23)	1	Histidine and derivative
Enoxolone	HMDB0011628	↓ feces (23)	1	Triterpenoids
H-arg-glu-OH	HMDB0028708	↓ feces (23)	1	Dipeptides

HMDB = www.hmdb.ca

↓, underrepresentation value in the biological product.

→, not decreased nor elevated value.

↑, overrepresentation value in the biological product.

AMR, antibody mediated rejection; TCMR, T, cell mediated rejections; ESKD, end stage kidney disease; KT-SKF, kidney transplant with stable kidney function; BKVN, BK virus nephropathy; n/a, not applicable.

Discriminative metabolites of *acute graft rejection after transplantation* were detected, including creatinine, kynurenine, uric acid, polyunsaturated fatty acid, phosphatidylcholines, sphingomyelins, lysophosphatidylcholines, etc. Zhao et al. investigated serum metabolite profile in 27 patients undergoing kidney transplant. Among these, 11 were diagnosed with acute rejection. The lower level of serum *dehydroepiandrosterone sulfate* was found in the acute graft rejection group compared to those who did not present graft rejection (24).

Sigdel et al. conducted a comprehensive investigation of metabolites in distinct kidney transplant complication, comparing acute rejection, polyoma BK nephropathy, interstitial fibrosis and tubular atrophy (IFTA) with stable transplants (STA). In acute rejection versus stable transplant, 146 metabolites were significantly altered (42 increased and 104 decreased). Augmentation of starch and *sucrose metabolism, galactose metabolism aminoacyl-tRNA biosynthesis, glutathione metabolism, and Glycine*, *serine and threonine metabolism* during acute rejection was observed (22).

In a study by Kalantari et al., a panel of nine differential metabolites encompassing nicotinamide adenine dinucleotide, 1-methylnicotinamide, cholesterol sulfate, gamma-aminobutyric acid (GABA), nicotinic acid, nicotinamide adenine dinucleotide phosphate, proline, spermidine, and alpha-hydroxyhippuric acid were identified as novel potential metabolite biomarkers of T-cell mediated rejection. *Proline, spermidine, and GABA* had the highest area under the curve (>0.7). Additionally, the study underline *nicotinamide and nicotinamide metabolism* as the most important pathways associated with T cell mediated rejection (18).

*The impact of immunosuppressive medication on the metabolite profile* was the main outcome of 10 studies totaling a number of 556 patients (32–41). Details about this outcome are reported in the **Table 5**. Kouidihi S et al. highlighted the impact of immunosuppressive therapy, revealing that the metabolomic signature showed dramatic changes. 21 metabolites were identified and they could mainly be classified into 9 fatty acids and long-chain fatty acids, 3 phenolic compounds, 2 amino acids, and 7 other classified metabolites. The identified metabolites mainly correspond to alterations of biosynthesis of *unsaturated fatty acids and tryptophan metabolism (* 49). Dieme et al. study offers helpful information into the urinary metabolomic profile of patients receiving *calcineurin inhibitors.* They identified different urinary metabolomic patterns at day 7, months 3 and 12 between the two groups treatment but also within each group over time. The principal metabolites presenting disparity levels between the two groups were mainly sugars, inositol, and hippuric acid (48). Similar information was offered by Le Guellec et al. using 1H-Nuclear Magnetic Resonance (NMR) and gas-chromatography/mass spectrometry (GC/MS) to profile urine samples of 47 kidney transplant patients, they identified specific metabolites, such as hippurate, lactate, and various sugars, that differentiated patients receiving TAC from those receiving CsA. Irrespective of the calcineurin inhibitor used, the urinary metabolite profiles differed between day 7 and month 3 or month 12 but was similar between month 3 and month 12 (49).

**Table 5:** Effect of immunosuppressive medication on metabolomic profile.

Author, Year	Participants, No	Specimen, No	Outcome	Results	Follow-up
1) Burghelea D. Et all, 2022 (32)	19 high levels TAC 23 low levels TAC	Blood - 1 per patient	Using machine learning+ metabolites to discern High vs Low levels of TAC.	All the metabolites that differed between the H-TAC and L-TAC groups were components of the lipid metabolism. Using a selected panel of five lipid metabolites, Mg2+, and uric acid, all three algorithms yielded excellent classification accuracies between the two groups.	May 2020-july 2020
2) Dieme B et all, 2014 (33)	TAC – 23 CsA - 12	Urine – at Day 7, M3 and M12	Analyze metabolic profile of CnI patients.	The urinary metabolic patterns varied over time in cyclosporine- and tacrolimus-treated patients and were different at D7, M3, M12 between the 2 treatment groups. Principal metabolites that differed, were mainly sugars, inositol, and hippuric acid.	12 months
3) He X. et all, 2022 (34)	109	Blood - 1 at day 7, M1, M3	Relationship between the pharmacodynamics and metabolic profiling,	Multinomial logistic regression analysis established a bridge that could quantify the relationship between the efficacy of tacrolimus and biomarkers. The results showed a good correlation between endogenous molecules and the efficacy of tacrolimus.	3 months
4) Kim CD. et all, 2010 (35)	57 – 27 - CsA 30 - TAC	Serum – Baseline – preTrx, M1, M3, M6	Metabonomics to integrate the serum metabolic profiles of transplant recipients with normal allograft function and identify time-dependent changes in the levels of serum metabolites in response to CsA- or TAC-based immunosuppression	The Partial Least Squares-Discriminant Analysis score plots showed a clear separation between levels at baseline and at 1, 3, and 6 months after KT in both groups.	6 Months
5) Kim S. Et all, 2013 (36)	20 – 3 SIR 8 TAC 9 HC	Blood	Utilized metabolomics to investigate a population of renal transplant patients maintained on a steroid-free monotherapy regimen of either sirolimus or tacrolimus.	False Discovery Rate analyses between HC, TAC, and SIR subjects revealed that N-acetylornithine and agmatine were, on average, lower in transplant patients relative to HC.	NR
6) Klepacki, J. Et all, 2016 (37)	120	Blood + Urine – baseline, M1, M2, M4, M6	Assess potential differences between the everolimus+ low-dose TAC and the MMF+ standard dose TAC treatment arms in the Novartis CRAD001AUS92 multicenter trial.	There were no significant differences in any of the other more than 600 evaluated parameters between the two treatment arms,. Except the effects directly associated with mycophenolic acid and everolimus mechanisms of action, there was no evidence for differences in immune, inflammation, vascular and kidney dysfunction markers between the two treatment arms.	6 Months
7) Le Guellec et all, 2013 (38)	47	Urine- Day 7, M3, M12	Used metabolomics to explore, whether the metabolic pattern differed according to the CNI used and if it varied over time	Metabolites that differentiate the drugs each others were hippurate, lactate and various sugars. Whatever the CNI used, the urinary metabolite profiles were shown to diverge between D7 and M3 or M12 but were not distinguishable between M3 and M 12. Metabolite proliles were different according to renal function at each time-point.	12 Months
8) Muhrez K. et all, 2014 (39)	38- 25- TAC 13- CsA	Urine – Day 7, M3, M12	Evaluated whether urine metabolomics may be used to monitor kidney function and discover new biomarkers in renal transplantation	Good discrimination between urine samples was obtained at different times, within each CNI group. No difference was found between patients with good or poor renal function at M3 and at M12. No relationship was found between metabolomic profiles at D7 and renal function at M3 or M12, indicating that the early profile may not help predicting later renal status.	12 Months
9) Xia F. Et all, 2018 (40)	299 11 – TAC nephrotoxicity 288 - HC	Urine	A targeted metabolomic assay to quantify 33 amino acids win patients with TAC nephrotoxicity	Analysis on urine from healthy volunteers and renal transplantation patients with tacrolimus nephrotoxicity confirmed symmetric dimethylarginine and serine as biomarkers for kidney injury, with AUC values of 0.95 and 0.81 in receiver operating characteristic analysis.	NR
10) Zhang F. Et all, 2018 (41)	66 – 42 – KT 24 – HC	Blood	Role of metabolomics in the identification process of potential biomarkers for acute kidney injury among the patients receiving renal transplantation	The most significant changes of the explored metabolites were related to the disturbance of tryptophan metabolism and arginine metabolism.	NR

KB, kidney biopsy; CsA, Cyclosporine A; TAC, Tacrolimus; M, Month; KT, Kidney Transplant; SIR, Sirolimus; HC, Healthy controls; MMF, mycophenolate mofetil; CNI, calcineurin; Inhibitor IGF, immediate Graft Function; DGF, delayed Graft Function; NR, not reported.

*Steroids*, one of the first classes of medication used in kidney transplantation, are a cornerstone in the treatment of kidney graft recpients (69). Considering the important side effects, efforts were made to develop a steroid free treatment regime that could provide valuable advantages in kidney transplant patients. Kim S et al. evaluated a population of steroid free patients with monotherapy with either Sirolimus or tacrolimus. They further explored metabolic differences between these patients and healthy controls. Their analysis revealed that N-acetylornithine and agmatine levels were, on average, lower in transplant patients compared with healthy controls. KEGG pathway mapping based on these metabolites showed that arginine and proline metabolism was significantly affected between the patient groups (36).

*Metabolomic approaches have been used to investigate the different alterations associated with opportunistic infections* (66). In the aforementioned study, Sigdel et al. compared metabolomic profile in patients with polyoma BK nephropathy versus stable kidney and founded that 90 metabolites were increased and 73 were decreased between groups. The variable selection using random forests methods generated a panel of four metabolites - *arabinose, 2-hydroxy-2-methylbutanoic acid, octadecanol, and phosphate*, that distinguish BKVN from stable kidney transplant. Detailed findings of the outcome analysis are presented in **Table 4**, summarizing the results of the included studies, and their implication for kidney transplant patients (51).

In addition to the categories presented above, we identified a subset of studies that did not fit in these categories, so we proceeded in labeling them as miscellaneous (16, 58–65, 68). These studies covered a mix of topics such as: metabolite profile evolution over time after kidney transplant (Gagnebin et al. (63)), metalomics in tubular injury (Wang J, et al. (65)), general profiling of kidney transplant recipients – blood, urine, saliva (Iwamoto et al. (16)). Even if these studies did not evaluate primary outcomes of interest, they provide valuable and detailed information into other aspects of metabolomics usage in kidney transplant patients, and contribute to the overall understanding of the topic. Details regarding outcome are provided in **Table 6**.

**Table 6:** Summary of other study outcomes.

Author, Year	Participants	Specimen, No	Outcome	Results	Follow up	KB
1. Calderisi, M et al. (58)	15 – 9 males and 6 females	Urine – at least 9 samples each	Kidney graft recovery process through the examinations of urine samples	Obs. 3 stages of recovery: initially the kidney is not working; in the second stage, it regains functions, and the third stage includes follow-up during hospitalization	NR	NR
2. Iwamoto, H. et al. (16)	59 KT impaired KF:31 KT stable KF:19 Donors:9	Blood Urine Saliva	Metabolomic analysis of plasma, urine, and saliva samples	In total, six metabolites showed different concentrations in the plasma (*p* < 0.05); In saliva samples, five metabolites showed significantly lower concentrations in TCR than in KD. Plasma and urine samples showed different metabolite patterns;	NR	Yes
3. Kienana M. et al. (59)	25 Tac 13 CsA	Urine 72: D7: 38 (25 Tac, 13 CsA) M3 and M12: 34 (22 Tac, 12 CsA)	The metabolite content of urines of renal transplant patients	Analysis of urine metabolomic profiles is a very useful method to study patho-physiological alterations in kidney transplant patients over time.	12 months	NR
4. Kouidhi S., et al. (60)	40 SG: 11 MG: 20 LG:9 20 HC	Fecal	Fecal metabolic signature of stable KT patients compared to healthy subjects.	Globally, the fecal metabolic signature was significantly different between kidney transplants and the control group	NR	NR
5. Li L., et al. (61)	20 KT 28 HC	Serum – 1 at time: HC – class 4 Before KT - class 1 1^st^ day after KT – class 2 7^th^ day after KT – class 3	Using Metabolomics to investigate the altered metabolic pattern in serum.	Compared with class 4, 19 different peaks and 10 potential biomarkers were identified in class 1, class 2, and class 3 (p < 0.0001).	7 days	NR
6. Stanimirova I., et al. (62)	19 KT	Serum T0 before KT T1 1^st^ day after KT T2 7^th^-10^th^ day after KT T3 6^th^ month after KT	Identify serum metabolites -> important when describing the shorter to longer-term (up to 6 months) graft accommodation	The changes in levels of hippurate, mannitol and alanine may be associated with the changes in renal function during the post-transplantation recovery period.	6 months	NR
7. Gagnebin Y et al. (63)	42 KT+ 24 donors	KT: G0 – before KT G1 – 1^st^ week after KT G2 – 1^st^ month after KT Donors: V0 – before KT V1- 1^st^ week after V2 – one year after	Impact of KT over time on the plasma metabolic profile of graft patients and donors.	Short and medium term benefits for patients with KT ( the blood metabolome remains relatively stable one month after transplantation This was also confirmed by a stable eGFR value, with no marked difference between G1 and G2) Relative low negative impact on donors.	NR	NR
8. Liu R et al. (64)	190 transplanted kidneys 35 disicarded kidneys (still evaluated by HMP)	Hypotermic machine perfusion of the kidney graft collected at the beginning and end of deceased-donor kidney	Measured all metabolites in perfusate and evaluated their associations with graft failure.	Metabolomics analysis: 629 unique metabolites:76 (unknown chemicals) and 165 (perfusate origin) were excluded from the analysis => 388 “de novo” metabolites. Metabolites significantly associated with dcGF: alpha-ketoglutarate, 3-carboxy-4-methyl-5-propyl-2-furanpropanoate, 1-carboxyethylphenylalanine, and three glycerolphosphatidylcholines. All six metabolites were associated with an increased risk of graft failure.	NR	NR
9. Ma C. et al (68)	NR	NR	NR	NR-not reported		
10 .Wang J. et al. (65)	10: 5 KT+ 5 donors	53 urine samples	Metabolomics usage for diagnosis of acute tubular injury after KT	ATI status during the recovery process of renal allografts after transplantation as changes among urine small molecule metabolites distinguished ATI/ATN	NR	yes

KT, kidney transplant; KF, kidney function; CsA, Ciclosporine A; Tac, Tacrolimus; HC, healthy control; dcGF, death-censored Graft Failure; ATI, acute tubular injury; ATN, acute tubular necrosis; NR, not reported.

In a population of 40 stable kidney transplant participants and 20 healthy controls Kouidhi S et al. (60) compared the fecal metabolic signature between these two cohorts. They observed variation in several metabolic pathways of Ubiquinone and other terpenoid-quinone biosynthesis, tyrosine metabolism, tryptophan biosynthesis and primary bile acid biosynthesis, due to immunosuppressive therapy. The study suggests that the tyrosine metabolism pathway may be predominantly linked with kidney transplantation period and immunosuppressive therapy. Additionally, it seems that there was no difference in endogenous metabolites between long-term and short-term post-transplant patients, indicating that these metabolic alterations do not appears to be strongly influenced by the length since transplant (60).

Studies that evaluated the pediatric population were identified and evaluated (26–31). 6 studies with a total of 537 patients were included. Outcomes can be found in **Table 7**.

**Table 7:** Studies evaluating pediatric kidney transplant recipients.

Author, Date	Participants	Specimen, No	Outcome	Results	Follow-up	KB
1) Sigdel T.K. et al., (26)	310	Urine - 326	AR	Post-Tx alloimmune injury: Glycine, N-methylalanine, Adipic acid, Glutaric acid, Inulobiose, Threitol, Isothreitol, Sorbitol, Isothreonic acid *Sensitivity = 95.3%, Specificity = 75.9%* Acute rejection of KTx: Glycine, Glutaric acid, Adipic acid, Inulobiose, Threose, Sulfuric acid, Taurine, N-methylalanine, Asparagine, 5-aminovaleric acid lactam, Myo-inositol *92.9% Sensitivity and 96.3% Specificity* BK virus nephritis: Arabinose, 2-hydroxy-2-methylbutanoic acid, hypoxanthine, benzyl alcohol, and N-acetyld-mannosamine *72.7% Sensitivity and 96.2% Specificity*	NR	yes
2) Archdekin B. et al., (27)	59	Urine - 396	Non-rejection KI	Top 20 metabolites contributing to the NRKI vs no NRKI dscore: Orn, Met.SO, C10.2, Leu, Hexose, Ac.Orn, PC.aa.C34.4, Pro, C5.1, C4, C3.OH, PC.ae.C44.5, PC.aa.C30.2, ADMA, Histamine, C9, Met, C2, C5.M.DC, C5.OH..C3.DC.M	NR	yes
3) Blydt-Hansen T. et al., (28)	57	Urine- 277 No TCMR – 183 Borderline Tublitis – 54 TCMR - 30	1) Non-invasive screening for TCMR. 2) Differentiate TCMR from no TCMR	3-component discriminant model for TCMR vs NR with auROC=0.89 The model for borderline vs NR had auROC=0.84 Five of the top 10 metabolites were common to both models. Urine metabolomics profiles provide robust non-invasive discrimination of TCMR and borderline tubulitis from a heterogeneous non-rejection phenotype.	NR	yes
4) Blydt-Hansen T. et al., (29)	57	Urine - 396	Urine metabolomic may provide biomarkers of AR	AMR is readily distinguishable from NR, AUC = 0.84 (95%CI 0.77- 0.91, p<0.01). Multivariate best-model selection identified DSA, biopsy indication and histological scores .To explore TCMR confounding, samples were restricted to TCMR present (n=148) or absent (n=235), and continued to differentiate AMR from NR, AUC=0.89 (95%CI 0.8-0.97, p=0.055) and AUC=0.83 (95%CI 0.73-0.93) respectively. Metabolite rankings by importance for these two classifiers were similar, indicating common metabolite perturbations from AMR in the presence/ absence of TCMR.	NR	yes
5) Blydt-Hansen T. et al., (30)	59	396 of which 40 - AMR 65 – Intermediate AMR 278 – No AMR	Urinary metabolomics for early noninvasive detection of AMR	Urine samples of patients with and without AMR were analyzed and a classifier for AMR was identified (P = 0.006) – based on the metabolomics	NR	yes
6) Taha C. et al. (31)	Urine	52	MMF exposure based on Metabolomics	Partial least squares (PLS) discriminate analysis was used to develop a top 10 urinary metabolite classifier that estimates MPA exposure. This urinary metabolite classifier can estimate MPA exposure and correlates with allograft inflammation.	NR	NR

KB, Kidney Biopsy; AR, acute rejection; TCMR, T-cell mediated rejection; KI, Kidney Injury; AMR, Antibody-mediated Rejection (Banff grade); NR, non AMR(Banff grade); IND, intermediate (Banff grade); AUC, area under curve; MMF, mycophenolate mofetil; DSA, donor-specific HLA antibodies; NR, not reported.

Sigdel et al. in a 2020 study, evaluated allograft disfunction caused by different causes. Using advanced data analysis techniques, including pattern recognition and selection, involving methodology that performed a rigorous scan of the dataset, they could conclude that a panel of 9 metabolites could be used accurately classify post-transplantation alloimmune injury; for acute rejection a metabolite marker panel of 11 metabolties could be used to detect acute rejection. As for BK virus nephropathy (BKVN) 5 metabolites were identified as BKVN-specific metabolites Arabinose, 2-hydroxy-2-methylbutanoic acid, hypoxanthine, benzyl alcohol, and *N*-acetyl-d-mannosamine). Pathway analysis for enrichment identified nitrogen metabolism, ascorbate and aldarate metabolism, and amino sugar and nucleotide sugar metabolism as the three most significantly enriched pathways (26).

Assessing the quality of the studies that were included in this review was realized with the Newcastle-Ottawa Scale (NOS); this tool is widely accepted as a method to evaluate and stratify the quality of methodology of included studies and the risk of bias in non-randomized studies. The scale evaluates 3 aspects of each study: the group selection, comparability and exposure. Each study receives points based upon fulfilling the criteria, with higher score indicating lower risk of bias. A detailed evaluation of each study based on NOS can be found in **Table 1**.

## 4 Discussion

The field of metabolomics is relatively new and continues to expand with rapidly emerging insights. Exploring findings from other areas, such as hematopoietic stem cell transplantation, can offer valuable strategies for application in kidney transplantation. A novel concept evaluated to gain deeper insights into a major complication afflicting allogeneic hematopoietic stem cell transplantation—graft-versus-host disease—could also hold potential application in kidney transplantation: metabolomic reprogramming. Metabolic pathways such as fatty acid oxidation and glycolysis play an important role in T cell function in the context of graft versus host disease. While the activity of pro-inflammatory effector T cells is driven by glycolysis, fatty acid oxidation plays a role in stabilizing regulatory T cells (Tregs). These results suggest a possible new strategy for preventing graft rejection. Combining an inhibition of glycolysis while simultaneously enhancing fatty acid oxidation to boost Treg stability, could lead to a more tolerant immune environment (70). Furthermore, Tomaszewicz M. et al., in a recent study, highlighted that targeted metabolic modulation could enhance Treg efficacy in promoting immune tolerance with a nuanced perspective: modulation toward a particular metabolic stage of Tregs that may improve or weaken cell stability and function. This approach could allow the adaptation of Treg responses based on specific needs -maintaining activation during infections or suppressing it when autoimmunity occurs (71).

Our systematic review underlines the substantial role of metabolomics in improving the understanding and management of kidney transplantation. The findings from the review offer several significant suggestions for clinical practice and future research.

### 4.1 Metabolomics as a tool for monitoring allograft function

The reviewed studies constantly establish that certain metabolites can function as consistent biomarkers for monitoring kidney allograft function. For example, elevated levels of glutamine and histidine have been linked with acute rejection, whereas a mixture of metabolites such as hippurate, mannitol, and alanine has been proposed as subtle indicators of renal function fluctuations. These biomarkers can possibly complete the customary methods such as serum creatinine and eGFR, delivering a more subtle interpretation of allograft status and possibly permitting a prompt intervention in cases of dysfunction.

Despite progresses in immunosuppressive regimens, rejection continues to be one of the biggest concerns confronted by both patients and their physicians. Prompt diagnosis and optimal management are essential for improving patients’ prognosis. Ultrasound-guided biopsies of the transplanted kidney are the contemporary gold standard for identifying rejection. However, hematoma, gross hematuria or hydronephrosis are sporadic but tangible complications. The ability to differentiate among several rejection types across metabolites such as creatinine, kynurenine, and sphingomyelins delivers a hopeful opportunity for personalized medicine. For example, several urine metabolites (Ribonic acid, glycolic acid, 3-hydroxyisovaleric acid, and octadecanoic acid) have been recognized as potential biomarkers to efficiently differentiate between acute renal allograft rejection and stable transplant recipients. Diagnostic sensitivity and specificity were determined as 80 and 86.7% respectively, with accurate diagnosis of 12 out of 15 renal allograft patients with acute rejection and 13 out of 15 patients with stable kidney function (25). Furthermore, a panel of nine difference metabolites encompassing nicotinamide adenine dinucleotide, 1-methylnicotinamide, cholesterol sulfate, gamma-aminobutyric acid (GABA), nicotinic acid, nicotinamide adenine dinucleotide phosphate, proline, spermidine, and alpha-hydroxy hippuric acid were distinguished as novel potential metabolite biomarkers of T cell mediated rejection (18). Another frequent complication of kidney transplantation that needs addressing represents ischemia-reperfusion injury. A severe condition that is mainly related to donor and recipient factors coupled with graft manipulation during storage. Further involving mechanisms such as oxidative stress, metabolic changes, cell death, microvascular damage and tubular damage are ultimately leading towards kidney damage: metabolomic studies could help establishing a nephroprotective strategy. Identifying pathways with specific molecular signature, metabolic check-points that could be further modulated through various interventions, represent just a few of the of the numerous possibilities metabolomics offers in uncovering novel therapeutic targets, refining precision medicine approaches, and deepening our understanding of complex biological systems (72).

Moreover, the dynamic nature of metabolomic variations following transplantation, as detected in studies by Stenlund et al. and Stanimirova I et al., underlines the value of constant monitoring. The proximate post-transplant period contains important metabolic shifts as the body adjusts to the new organ; several metabolites such as valine, alanine, glutamine, methionine, GPC (glycerophosphocholine) + APC, mannitol, glucose and lower levels of creatinine, citrate, myo-inositol, lactate, histidine, hippurate and adenine had relatively higher levels compared to the metabolite levels that were determined before transplantation. Additionally, the variations in levels of hippurate, mannitol and alanine may be related with the variations in renal function during the post-transplantation recovery period. Precisely, the level of hippurate (or histidine) is more sensitive to any short-term changes in renal activity than creatinine (62). These findings emphasize the need for ongoing metabolomic assessment to seizure the developing metabolic landscape and address possible complications proactively.

### 4.2 Pediatric considerations and miscellaneous findings

The subdivision of studies concentrating on pediatric patients and additional various topics, for instance the influence of tubular injury and general metabolite profiling, supplements the complete understanding of metabolomics in kidney transplantation. The pediatric population exhibits unique challenges in the setting of kidney transplantation and management. Medication dosing, interactions with the growth factors that are specific to this age and different particular pathophysiologic pathways pose a unique twist in this category. Also, in this population a defined urinary metabolite profile was correlated with T cell mediated rejection; 10 urinary metabolites, including Proline, Kynurenine, and Sarcosine, were noticed to be the most important; of these metabolites, 5-10 appeared to be shared with borderline tubulitis, advocating that allograft injury associated with the T cell-mediated alloimmune response may occur on a continuum of severity. A urinary metabolite signature associated with non-rejection kidney injury in pediatric kidney transplant recipients was also investigated in a single-center pediatric cohort study (28). 20 quantified urinary metabolites were associated with kidney injury independent of acute rejection; of these, proline, ADMA, urinary hexose, butyrylcarnitine, acetylcarnitine and non-acylcarnitine were associated with inflammatory injury. Increased urinary acylcarnitines have also been recognized in patients with diabetic kidney disease who have developed micro- or macroalbuminuria. Urinary carnitine may insinuate mitochondrial and proximal tubule injury. Each of these metabolites makes a relative influence to discrimination, but it is the patterned modification in metabolism characterized by all of these metabolites collectively that delivers robust discrimination (27).

While the outcomes were categorized broadly into areas such as kidney rejection, immunosuppression, and graft function, which might facilitate the aggregation of results and their interpretation, the variability across studies concerning design, patient demographics such as underlying conditions, the age of kidney transplants, as well as how outcomes were defined, measured, and reported, created considerable difficulties in quantitatively combining the results. Moreover, the studies referenced in our paper were typically examining different metabolites that were not directly comparable across the various studies.

## 5 Limitations and future directions

We can highlight some strong points regarding our review. We performed an extensive database coverage, ensuring thus that our review included studies from multiple databases, such as the ones listed above; we also included conference abstracts, reports avoiding thus some publication bias or missing some unpublished articles that may have had significant findings. We have precise criteria for selecting studies resulting thus in consistency and transparency. We utilized tools for assessing the quality of our included studies (Newcastle-Ottawa Scale).

While the review underlines promising opportunities for metabolomic uses in kidney transplantation, some limitations demand attention. Despite the efforts made that the bibliographical search would be as complete as possible, with double checking the databases for an exhaustive process, some articles, relevant to the topic, may have not been found and therefore omitted from this review. An additional limitation is the English language, as we included studies that were written primarily in English. This may have excluded other potential relevant studies published in other languages, thus having a potential impact on our findings The heterogeneity in study designs, missing demographic data, sample sizes, and metabolomic techniques are challenges in normalizing findings. In addition to these challenges, missing information could impact the quality assessment considering that the NOS has its limitations Moreover, the cross-sectional nature of many studies limits the ability to establish causal relationships between metabolomic changes and clinical outcomes. Future research should focus on larger, multicenter longitudinal studies to validate identified biomarkers and explore their clinical utility in routine practice with an emphasis on enhance methodological consistency that is needed to facilitate better synthesis and interpretation of findings.

## 6 Conclusion

In conclusion, our systematic review underlines the possible role of metabolomics in monitoring kidney transplant patients. By integrating data from numerous studies and analyzing metabolomic profiles in blood, urine, and fecal samples, we have detected possible new biomarkers that might transform the method we screen kidney transplant recipients. Including other ‘omics’ technologies, such as genomics and proteomics, could extend our consideration of the molecular mechanisms behind graft rejection and dysfunction. This multi-omics approach could deliver complete insights into the biological procedures involved.

## Data Availability

The original contributions presented in the study are included in the article/**Supplementary Material**. Further inquiries can be directed to the corresponding author/s.
