# Supplementary material for: Exploring the role of metabolomics in kidney transplantation: a systematic review of the literature
Source: Front Immunol. 2025 Jun 10;16:1534875. doi: 10.3389/fimmu.2025.1534875 (PMC12186729; doi:10.3389/fimmu.2025.1534875)
Supplement: Supplementary file 2 [file Table2.docx]

**Identification of studies**

Records removed *before screening*:

Duplicate records removed (n = 1115)

4549 Records identified from:

PubMed (n=3029)

*Embase* (n=1514)

Cochrane (n=6)

**Identification**

Records screened

(n = 3434)

Records excluded by title/abstract

(n =3323 )

**Screening**

53 Reports excluded:

Not in English (n=2)

Included animal models (n=4)

Non-metabolomic focus (n=25)

Ineligible study designs (n=22)

Reports assessed for eligibility

(n =111 )

Studies included in review

(n = 58)

**Included**

Source: Page MJ, et al. BMJ 2021;372:n71. doi: 10.1136/bmj.n71.
