# Supplementary material for: Exploring the role of metabolomics in kidney transplantation: a systematic review of the literature
Source: Front Immunol. 2025 Jun 10;16:1534875. doi: 10.3389/fimmu.2025.1534875 (PMC12186729; doi:10.3389/fimmu.2025.1534875)
Supplement: Supplementary file 3 [file Table3.docx]

| Author, Year | Participants | Specimen, No | Outcome | Results | Follow-up | KB |
| --- | --- | --- | --- | --- | --- | --- |
| 1) Alkadi M. et. All. – 2016 ^1^ | ATI – 56 NKF – 29 AMR- 20 | Urine  61 - ATI  60 – NKF  20 – AMR | AMR | Osmolarity-corrected 3SL, QUIN, and X-16397 were significantly higher in the AMR Group than in the normal group (P values 0.02, <0.0001, and 0.05, respectively). Osmolarity-corrected QUIN was also significantly higher in the AMR Group than in the ATI Group (P = 0.02). The ratio of QUIN/X-16397 distinguished the AMR group from the Normal Group (P = 0.006) and AMR group from the ATI Group (P = 0.003) | NR | Yes |
| 2) M. Banas et. All – 2018 ^2^ | 178 | Urine – 589 | AR | Core model of alanine, citrate, lactate to which a few models added urea, glucose and/or glucuronate to the core set. In order to estimate the expected classification performance, we generated ROC curves and computed the area under the curve as a measure of performance - for the test set the observed values were 0.72 and 0.74 for the strict and extended setting. | 12 months | yes |
| 3) M banas et all – 2018 ^3^ | 109 | Urine -2479 | AR | The metabolic model was able to detect acute cellular allograft rejection during outpatient phase (≥ day 15 after transplantation, AUC 0.75, [95% confidence interval (CI) 0.68 to 0.83], P < 0.001). A combination of the test with serum creatinine based eGFR significantly improved the overall performance AUC 0.84. | 12 months | yes |
| 4) Dedinska I et. All. – 2022 ^4^ | 55 | Blood | AR | From 23 metabolites:  - Lactate was increased in CG and TCMR vs AMR;  - Plasma Glutamine decreased in CG against TCMR and AMR;  - Thyrosine increased in CG against AR | Average 68,4 months  Median 59 months | yes |
| 5) Iwamoto et. All. – 2022 ^5^ | Healthy -9  NKF after KTR – 19  Impaired renal after KTR – 32 | Blood  Urine  Saliva 60 | AR | In the group of subjects with impaired kidney function after kidney transplantation 8 substances in blood, 3 substances in urine and 7 substances in saliva that were significantly different (p<0.001) from those in the control group, the normal kidney function after kidney transplantation, and the group of kidney transplant donors | NR | yes |
| 6) Iwamoto et all – 2018 ^6^ | KTR with High creat – 32  KTR w/o high creat – 19  Healthy – 9 | Saliva | AR | Various metabolites showed clear difference among these three groups. Seven metabolites: 2-hydroxyglutarate, adipate, ethanolamine phosphate, fumalate, glycolate, proline, sedoheptulose 7-phosphate showed significant difference (P < 0.001) between the groups (1) and (3) | NR | yes |
| 7) Kalantari s et. All. – 2020 ^7^ | 7 – TCMR  15 – IKF after KTR  6 SKF after KTR | Urine | TCMR | In all, a panel of nine differential metabolites were identified as novel potential metabolite biomarkers of TCMR. Proline, spermidine, and GABA had the highest area under the curve (>0.7) and were overrepresented in the TCMR group. Nicotinate and nicotinamide metabolism was the most important pathway in TCMR. | NR | yes |
| 8) Kim S et all – 2019 ^8^ | 385 total  TCMR vs | Urine | TCMR | ROC curve analysis showed the best performance of the training set (area under the curve value, 0.926; sensitivity, 90.0%; specificity, 84.6%) using a panel of five potential biomarkers: guanidoacetic acid, methylimidazoleacetic acid, dopamine, 4-guanidinobutyric acid, and L-tryptophan. | NR | yes |
| 9) Li X. et all – 2022 ^9^ | 60 – 28 with AMR si 32 control normal eGFR | Fecal 60 | AMR | 11 metabolites up-regulated, and 21 metabolites down-regulated in fecal samples from recipients with AMR. | NR | yes |
| 10) Mao y. et all -2008 ^10^ | 22 reject 15 stable | Serum | AR | 46 endogenous metabolites were identified in 37 recipients. Principal component analysis based on these metabolites discriminated acute rejection group from stable recipients., the levels of 17 metabolites were significant higher in rejection group than those in stable group. | NR | yes |
| 11) Sigdel t et all – 2018 ^11^ | n/a | Urine 308 | GD | - 266 metabolites identified -146 metabolites were significantly altered (42 increased and 104 decreased) in AR compared to STA.  - BKVN versus STA comparison, 163 metabolites (90 increased and 73 decreased).  - 128 metabolites (91 increased and 37 decreased) were significantly altered in the IFTA urine samples compared to the AR samples. | NR | NR |
| 12) Wang J. et all - 2023 ^12^ | 86 – 30 AMR, 35 stable function 21 ESRD | Fecal | AMR | Notably, there were significantly higher levels of N-Palmitoylsphingosine and Erucamide, and lower levels of 3b-Hydroxy-5-cholenoic acid, N-Acetyl-L-Histidine, Enoxolone and Arg-Glu in the KT-AMR group than the other groups. | NR | NR |
| 13) Zhao x et al – 2014 ^13^ | 27 – 11 AR, 16 non-acute Rejection | Serum | GR | Discriminative metabolites of acute graft rejection after transplantation were detected, including creatinine, kynurenine, uric acid, polyunsaturated fatty acid, phosphatidylcholines, sphingomyelins, lysophosphatidylcholines, etc. | NR | NR |
| 14) Zheng L. et all. – 2018 ^14^ | 30: 15 ARt+ 15 SKF | Urine | AR | Overall, 14 metabolites were significantly altered in the acute rejection group (11 and 3 metabolites displayed higher and lower levels, respectively) relative to the stable transplant group. | NR | NR |

AMR – antybody mediated rejection AR – acute rejection GR- graft rejection GD – graft dysfunction KTR – kidney transplant

TCMR – T cell mediated rejection ATI – acute tubular injury NKF – normal kidney function KB – kidney biopsy

eGFR – estimated glomerular filtration rate AUC – area under the curve ROC- receiver operator characteristic MKF – modified kidney function

SKF – stable kidney function IRF – impaired kidney function NR – not reported

1. Alkadi M, Lee J, Dadhania D, Muthukumar T, Snopkowski C, Li C, et al. Urine cell-free supernatant metabolites diagnostic of antibody mediated rejection in kidney allografts [abstract]. American Journal of Transplantation. 2016;16:247-8. Available at: [https://atcmeetingabstracts.com/abstract/urine-cell-free-supernatant-metabolites-diagnostic-of-antibody-mediated-rejection-in-kidney-allografts/.](https://atcmeetingabstracts.com/abstract/urine-cell-free-supernatant-metabolites-diagnostic-of-antibody-mediated-rejection-in-kidney-allografts/)
2. Banas M, Neumann S, Eiglsperger J, Schiffer E, Putz FJ, Reichelt-Wurm S, et al. Identification of a urine metabolite constellation characteristic for kidney allograft rejection. Metabolomics. 2018 Aug;14(9):116. doi: 10.1007/s11306-018-1419-8.
3. Banas M, Neumann S, Pagel P, Chittka D, Pfahlert V, Banas B. Detection of renal allograft rejection by NMR-based urine metabolomics [abstract]. Nephron. 2018;139(1):108.
4. Dedinska I, Baranovičová E, Graňák K, Vnučák M, Beliančinová M, Mokáň M. Metbolomics approach and acute rejection in kidney transplant reciepnts. Nephrology Dialysis Transplantation. 2022 May;37(3). doi:10.1093/ndt/gfac087.038
5. Iwamoto H, Konno O, Kihara Y, Okihara M, Akashi I, Ueno T, et al. Diagnosis of Acute Graft Rejection After Kidney Transplantation Using Metabolome Analysis by Liquid Biopsy Approach [abstract]. American Journal of Transplantation. 2022;22:1033. Available at: [https://atcmeetingabstracts.com/abstract/diagnosis-of-acute-graft-rejection-after-kidney-transplantation-using-metabolome-analysis-by-liquid-biopsy-approach/.](https://atcmeetingabstracts.com/abstract/diagnosis-of-acute-graft-rejection-after-kidney-transplantation-using-metabolome-analysis-by-liquid-biopsy-approach/)
6. Iwamoto H, Sugimoto M, Konnno O, Kihara Y, Yokoyama T, Nakamura Y, et al. Diagnosis of acute renal rejection using saliva metabolome analysis [abstract]. American Journal of Transplantation. 2018;18:958. Available at : [https://atcmeetingabstracts.com/abstract/diagnosis-of-acute-renal-rejection-using-saliva-metabolome-analysis/.](https://atcmeetingabstracts.com/abstract/diagnosis-of-acute-renal-rejection-using-saliva-metabolome-analysis/. )
7. Kalantari S, Chashmniam S, Nafar M, Samavat S, Rezaie D, Dalili N. A Noninvasive Urine Metabolome Panel as Potential Biomarkers for Diagnosis of T Cell-Mediated Renal Transplant Rejection. OMICS. 2020 Mar;24(3):140-147. doi: 10.1089/omi.2019.0158.
8. Kim SY, Kim BK, Gwon MR, Seong SJ, Ohk B, Kang WY, et al. Urinary metabolomic profiling for noninvasive diagnosis of acute T cell-mediated rejection after kidney transplantation. J Chromatogr B Analyt Technol Biomed Life Sci. 2019;1118-1119:157-63. doi: 10.1016/j.jchromb.2019.04.047.
9. Li X, Li R, Ji B, Zhao L, Wang J, Yan T. Integrative metagenomic and metabolomic analyses reveal the role of gut microbiota in antibody-mediated renal allograft rejection. J Transl Med. 2022;20(1):614. doi: 10.1186/s12967-022-03825-6.
10. Mao YY, Bai JQ, Chen JH, Shou ZF, He Q, Wu JY, et al. A pilot study of GC/MS-based serum metabolic profiling of acute rejection in renal transplantation. Transpl Immunol. 2008 Apr;19(1):74-80. doi: 10.1016/j.trim.2008.01.006.
11. Sigdel T, Yang J, Sarwal M. A “Multi-Omic” Analysis of Proteome and Metabolome for Acute Rejection and PVAN in Kidney Transplantation [abstract]. American Journal of Transplantation. 2017;17(3) Available at: <https://atcmeetingabstracts.com/abstract/a-multi-omic-analysis-of-proteome-and-metabolome-for-acute-rejection-and-pvan-in-kidney-transplantation/>.
12. Wang J, Zhang X, Li M, Li R, Zhao M. Shifts in Intestinal Metabolic Profile Among Kidney Transplantation Recipients with Antibody-Mediated Rejection. Ther Clin Risk Manag. 2023 Mar;19:207-17. doi: 10.2147/TCRM.S401414.
13. Zhao X, Chen J, Ye L, Xu G. Serum metabolomics study of the acute graft rejection in human renal transplantation based on liquid chromatography-mass spectrometry. J Proteome Res. 2014 May;13(5):2659-67. doi: 10.1021/pr5001048.
14. Zheng L, Wang J, Gao W, Hu C, Wang S, Rong R, et al. GC/MS-based urine metabolomics analysis of renal allograft recipients with acute rejection. J Transl Med. 2018;16(1):202. doi: 10.1186/s12967-018-1584-6.
