# Supplementary material for: Exploring the role of metabolomics in kidney transplantation: a systematic review of the literature
Source: Front Immunol. 2025 Jun 10;16:1534875. doi: 10.3389/fimmu.2025.1534875 (PMC12186729; doi:10.3389/fimmu.2025.1534875)
Supplement: Supplementary file 4 [file Table4.docx]

| Title, Year | Participants | Specimen, No | Outcome | Results | Follow-up | KB |
| --- | --- | --- | --- | --- | --- | --- |
| 1) Dadhania D. Et all, 2016 ^1^ | 62 –  29 Normal KB  33- BKVN | Urine – 107  33- Initial BKVN date  14 – follow-up | ratios of 3-sialyllactose to xanthosine (3-SL/X) and quinolinate to X-16397 (Q/X-16397) distinguish patients with BKV virus | The ratio of Q/X-16397 is significantly higher in the 33 cell free urine supernatants collected at the time of initial BKVN diagnosis compared to the Q/X- 16937 ratio in the 60 cell free urine supernatants collected from the 29 patients with normal biopsy results. | NR | Yes |

KB – Kidney Biopsy BKVN – BK virus nephropathy NR – not reported

# Bibliography

1. Dadhania D, Lee J, Alkadi M, Muthukumar T, Snopkowski C, Li C, et al. Urine metabolomics: Quinolinate, a product of tryptophan metabolism, is associated with BK virus nephropathy and intragraft inflammation in kidney transplant recipients. Transplantation. 2016;100(7):S609-S10.
